# Automatic diagnosis of COVID-19 infection based on ontology reasoning

**DOI:** 10.1186/s12911-021-01629-0

**Published:** 2021-11-16

**Authors:** Huanhuan Wu, Yichen Zhong, Yingjie Tian, Shan Jiang, Lingyun Luo

**Affiliations:** 1 School of Computer Sciences, University of South China, 28 West Changsheng Rd, Hengyang, 421001 People’s Republic of China; 2 Hunan Medical Big Data International Science and Technology Innovation Cooperation Base, Hengyang, 421001 People’s Republic of China

**Keywords:** COVID-19, Ontology, Diagnostic rules, SWRL rules, Automated diagnosis

## Abstract

**Background:**

2019-nCoV has been spreading around the world and becoming a global concern. To prevent further widespread of 2019-nCoV, confirmed and suspected cases of COVID-19 infection are suggested to be kept in quarantine. However, the diagnose of COVID-19 infection is quite time-consuming and labor-intensive. To alleviate the burden on the medical staff, we have done some research on the intelligent diagnosis of COVID-19.

**Methods:**

In this paper, we constructed a COVID-19 Diagnosis Ontology (CDO) by utilizing Protégé, which includes the basic knowledge graph of COVID-19 as well as diagnostic rules translated from Chinese government documents. Besides, SWRL rules were added into the ontology to infer intimate relationships between people, thus facilitating the efficient diagnosis of the suspected cases of COVID-19 infection. We downloaded real-case data and extracted patients’ syndromes from the descriptive text, so as to verify the accuracy of this experiment.

**Results:**

After importing those real instances into Protégé, we demonstrated that the COVID-19 Diagnosis Ontology showed good performances to diagnose cases of COVID-19 infection automatically.

**Conclusions:**

In conclusion, the COVID-19 Diagnosis Ontology will not only significantly reduce the manual input in the diagnosis process of COVID-19, but also uncover hidden cases and help prevent the widespread of this epidemic.

## Background

With the outbreak of the 2019-nCoV [1], the sharp increase in the number of COVID-19 infection cases has made medical supplies in short. Since the causes of COVID-19 are complex and diverse, the determination of suspected or confirmed cases of COVID-19 infection is quite time-consuming and labor-intensive. As a result, some cases were missed thus not reported, which would cause further infections. However, ontology is an abstract knowledge modeling, which treats the knowledge as concepts, associated attributes, and relations. On the other hand, according to the “Chinese Clinical Guidance for COVID-19 Pneumonia Diagnosis and Treatment (7th edition) [2, 3]” issued by the National Health Commission [4], the diagnostic criteria for suspected cases and confirmed cases is clearly and strictly defined, which makes the automatic diagnosis based on sufficient structured data possible. As such, to fill in the gap between documented rules and automatic diagnosis, this study constructed a COVID-19 Diagnosis Ontology (CDO) using Protégé [5], which considers the basic knowledge structure of COVID-19, as well as rules for automatically diagnosing suspected cases and confirmed cases of COVID-19 infection based on real patient data.

Furthermore, a key factor that affects the diagnosis of suspected cases of COVID-19 infection is the interaction between people. Thus, the collection of the social network among the crowd is inevitable for epidemiological analysis. In this study, we leveraged and expanded the lightweight social network ontology FOAF (Friend of a Friend) [6] towards capturing the intimate contacts between targeted people. The FOAF project is the earliest attempt by Libby Miller and Dan Brickley to introduce the Semantic Web into the field of social networks. It involves linking information to represent social networks, representational networks, and information networks. Through the expansion of the FOAF ontology and the usage of SWRL rules, The implicit kinship and contacts among people can be inferred, and get a more complete map of the social network around them, thus make it less likely to misjudge cases of COVID-19 infection.

To validate the performance of CDO, we collected the epidemiological survey data of COVID-19 patients in Ningbo, Zhejiang from the Health Commission of Ningbo [7]. Since the original data is unstructured, we performed pre-processing and obtained the structured characteristics of the patients, including epidemiological history, clinical manifestation, and examination results. Experiment results demonstrated that CDO showed good performances to automatically diagnose confirmed and suspected cases of COVID-19 infection based on real patient data, provided that the given information is sufficient.

## Related work

Since the outbreak of the 2019-nCoV, there have been many relative studies based on knowledge graphs. Zhang et al. [8] collected entities and relationships (including diseases, people, symptoms, etc.) related to COVID-19 from online texts, and constructed the COVID-19 Concept Knowledge Graph. Xu et al. [9] proposed a Health Knowledge Graph based on COVID-19 related diseases, drugs, symptoms, etc. The Aminer [10] team of Tsinghua University collaborated with multiple research teams and institutions and built a large-scale, structured knowledge graph of COVID-19 named COKG-19 [11], which covers aspects including but not limited to medical care, health, materials, prevention, and scientific research. COKG-19 aims to help researchers identify and link semantic knowledge in texts, and provide more intelligent services and applications to the users. The CIDO ontology (Ontology of Coronavirus Infectious Disease) [12] proposed by He et al. [12] is part of the OBO Foundry Ontology Library, which covers multiple areas in the domain of coronavirus diseases. CIDO is focused on analyzing COVID-19 from a medical standpoint. E.g., similarity to other viruses, common symptoms, etc. Although the knowledge graphs mentioned above are fairly comprehensive and provide an important theoretical basis for our research, they remain at the most basic level, and do not include rules for COVID-19 diagnosis.

CODO (COVID-19 Ontology for cases and patient information)[13] is an ontology that represents COVID-19 case data, which provides a model for the collection and analysis of data about the COVID-19 pandemic, such as identifying potential additional contacts who may be at risk due to their relationship with infected individuals. CODO divides confirmed cases into mild, moderate and severe cases, and its judgment rules are relatively simple, which may not be comprehensive enough for our tasks. In this study, we not only enriched the diagnosis rules for confirmed and suspected cases, but also defined rules to automatically achieve people’s travel history and residence history in high-risk areas, etc.

Chen et al. [14] from the Information Engineering University constructed a knowledge graph of COVID-19 by analyzing the activities of the population infected by COVID-19 according to the “5W1H” model [15] and integrating the existing general event representation model SEM [16]. They analyzed the transmission relationship between specific cases at the individual level and accurately located the transmission path of COVID-19, to provide technical support for medical staff in disease prevention and control. However, they analyzed the transmission relationship based on the patient’s activities under the condition that the patient was known to be a COVID-19’s confirmed case. Same as COKG-19, it did not focus on the diagnosis process as we did in this study.

## Methods

### Construction of the COVID-19 Diagnosis Ontology (CDO)

The most widely used tool Protégé [5, 17] will be used to encode the COVID-19 Diagnosis Ontology in this study. The main reasoner implemented was Pellet [18], which could obtain the inferred relationships and information by utilizing existing rules and data.

**Fig. 1:**
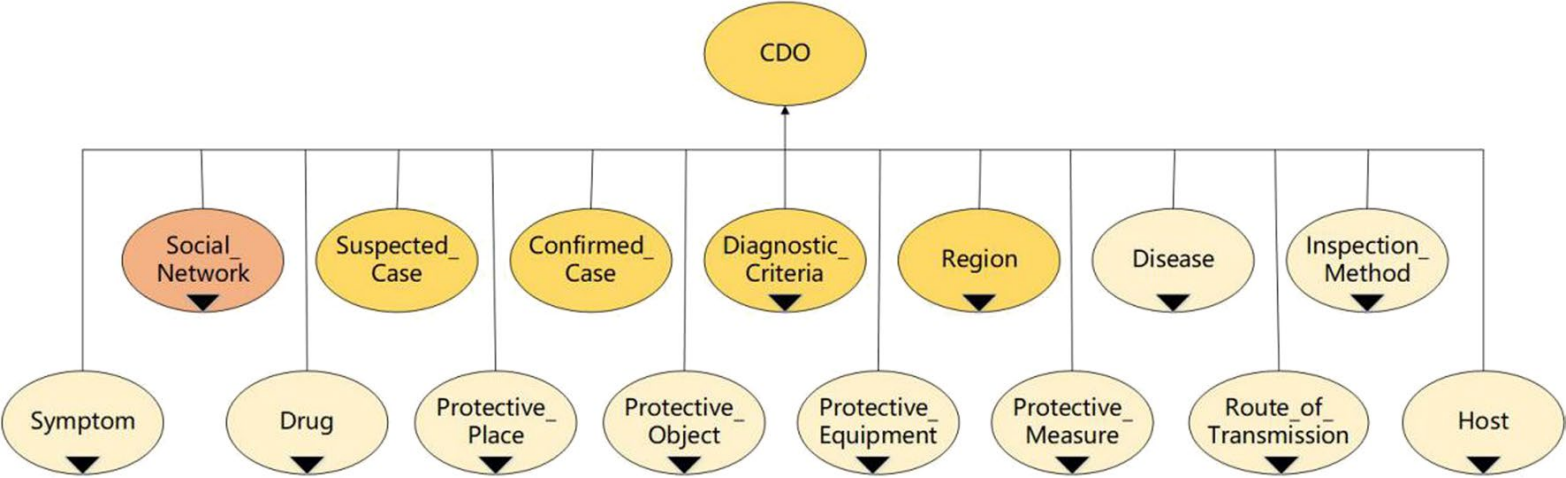
The hierarchical structure of the main classes in CDO, where the “inverted triangle” means that the class has subclasses

After analyzing the knowledge structure of COVID-19, we selected COKG-19 [11] as the basic knowledge base of our diagnosis ontology, on top of which, we added several classes especially for diagnosis purposes, and constructed CDO. The highest layer of CDO has a total of 15 classes (as shown in Fig. 1), among which 10 classes came from COKG-19 [11], including “Disease,” “Inspection_Method,” “Symptom,” “Drug,” “Protective_Place,” “Protective_Objects,” “Protective_Equipment,” “Protective_Measure,” “Route_of_Transmission,” and “Host.”

In addition, to capture the contacts between people, we also expanded the “Person” subclass from FOAF [6, 19] and created a new class named “Social_Network.” Also, the class “Diagnostic_Criteria” was added to demonstrate the diagnostic criteria according to Chinese government documents [2, 3]. The class “Region” was also added to decide the risk level of different places and to be further used for residence history and travel history analysis needed in the COVID-19 diagnosis process.

At last, we created two classes “Suspected_Case” and “Confirmed_Case” defined with restriction rules according to the diagnostic criteria for suspected and confirmed cases of COVID-19 infection respectively. Individuals that satisfy the restriction rules will automatically be classified as instances of the corresponding class.

#### Construction of the class “Social_Network”

COVID-19 is easy to be transmitted through personal contact with the extremely contagious of it in consideration. Given this, we created the class “Social_Network” in CDO by enriching the subclass “Person” from FOAF, aiming to derive social interaction and relationships among targeted people, so as to predict the probability of a person getting infected by COVID-19 through interaction with other people. Mainly, we expanded the subclass “Person” from two aspects. Firstly, we defined 14 relationships between instances of “Person” as object properties [20] using actual interpersonal relationships in society. The main object properties added to the ontology include *hasParent*, *hasSpouse*, *hasChild*, *hasUncle,* etc. The domains and ranges of all the object properties are both “Person.” In addition, we set characteristics [20] for object properties based on actual situations. For example, we set the property *hasSpouse* as “Symmetric”, and set *hasParent* and *hasChildren* as “Inverse functional.” After setting the relationships between instances, we can infer whether there is an infection based on the intimate relationships between people. For example, if a patient is a confirmed case, then it can be deduced that his spouse or child has a high probability of being infected too.

Leveraging the object properties defined in the class “Person,” we are able to provide the interpersonal relationships among instances. However, it is impractical to include every relationship into the ontology manually as people’s social networks are usually very complex. As a result, we introduced the Semantic Web Rule Language (SWRL) [21] rules to infer the implicit relationships that were not listed in the ontology. A total of 34 SWRL rules are available in the CDO, among which 24 were used to infer the implicit relationships between individuals (Please see Appendix Table 3). For example, the following rule (1) is an SWRL rule expressing that under the premise that x has brother y and father z, it can be inferred that y also has father z.

1 $$\text{hasBrother} (?\text{x}, ?\text{y})\wedge \text{hasFather}(?\text{x},?\text{z}){-}{>}\text{hasFather}(?\text{y},?\text{z})$$

**Fig. 2:**
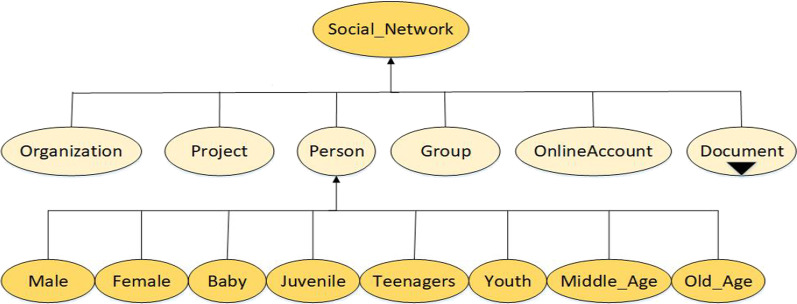
Hierarchy of the “Social_Network” class

Secondly, considering the differences in age and physiological function of each person, the chances of people getting infected by COVID-19 are different. It is possible to analyze the degree of human susceptibility to COVID-19 in different age groups by classifying confirmed cases into different age groups. Therefore in CDO, we further enriched the class “Person” by dividing the life of a person into 6 stages according to age: (1) Baby: 0 (newborn)–6 years old; (2) Juvenile: 7–12 years old; (3) Teenagers: 13–17 years old; (4) Youth: 18–45 years old; (5) Middle age: 46–69 years old; (6) Old age: > 69 years old [22]. The hierarchy of the classes is shown in Fig. 2.

Each stage is represented by the expression “Person and (age some xsd: integer[> = n])” [20]. For example, we use expression (2) to define the class “Old_Age,” similarly for other cases.

2 $$\text{Person and (age some xsd: integer)}[>=70])$$

For any age entered into CDO, it will be automatically recognized and classified as an instance of the corresponding age class. As a result, the cumulative number of confirmed cases of different ages can be collected for statistical analysis, so we can further analyze the degree of susceptibility to COVID-19 of different age groups.

### Automatic diagnosis of suspected cases and confirmed cases of COVID-19 infection

After the classes “Confirmed_Case” and “Suspected_Case” were defined, we need to set the sufficient and necessary conditions [20] for them in accordance with the diagnostic criteria for COVID-19. According to the “Chinese Clinical Guidance for COVID-19 Pneumonia Diagnosis and Treatment (7th edition)” [2, 3] issued by the National Health Commission [4], the diagnostic criteria for COVID-19 are mainly consist of three parts: epidemiological history, clinical manifestation, and etiological or serological examination. To be able to perform automatic diagnosis, we need to model the diagnostic criteria and translate them into classes, properties, and rules that are elements of the diagnosis ontology CDO.

#### Modeling of “Epidemiological History”

As stated in the document [3], epidemiological histories include 4 types:

1. Travel or residence history in Wuhan and surrounding areas, or other communities with documented COVID-19 positive cases within 14 days before the onset of illness.

2. History of contact with COVID-19-infected persons (positive for nucleic acid detection) within 14 days before the onset of illness.

3. History of contact with the patients presenting fever or respiratory symptoms, who travel to or reside in Wuhan and surrounding areas, or other communities with documented COVID-19 positive cases within 14 days before the onset of illness.

4. Clustering onset (2 or more cases of fever and/or respiratory symptoms within 2 weeks in small areas such as home, office, school class, etc.).

To determine whether a patient is a suspected case or a confirmed case of COVID-19 infection, it is necessary to analyze whether the patient meets one or more than one type of epidemiological history. Therefore, we model the epidemiological histories as data properties [20] on the class “Person” in CDO at first. Totally, we added 6 data properties of this kind: *travel history in a high-risk area* (referred to as *A1* in the remaining, similarly for other data properties, please see Appendix Table 4 for labels of data properties)*, residence history in a high-risk area(A2), contact with patients with positive nucleic acid tests(A3), contact with patients with a fever symptom(A4), contact with patients with a respiratory symptom(A5), clustering onset(A6).* The value of each data property is represented by 1 or 0, where 1 represents a positive value and 0 represents a negative value. For example, if someone has a travel history in a high-risk area, the value of *A1* will be 1.

As travel history is concerned, the above example is for the scenario when we know that a person had passed through a high-risk area, but do not know which specific place he had passed. Another scenario is indirect: we have the information on the regions that someone had passed, and want to use this information and SWRL rules to deduce whether he has *travel history in a high-risk area (A1).* To achieve this goal, firstly, we added a class “Region” to CDO and added three subclasses to it: “high_risk_area,” “medium_risk_area” and “low_risk_area.” Secondly, we added an object property *pass_by* from the class “Person” to the class “Region” to indicate the specific places a person had passed. Lastly, we also created a class named “Travel_History_in_High_risk_area” and used the SWRL rule (please see rule (3)) to define it.

3 $$\text{Person}(?\text{x})\wedge \text{pass}\_\text{by} (?\text{x},?\text{y})\wedge\text{high}\_\text{risk}\_\text{area}(?\text{y})->\text{Travel}\_\text{History}\_\text{in}\_\text{High}\_\text{risk}\_\text{area}(?\text{x})$$

This rule indicates that if x has passed through y, and y is a high-risk area, then we can conclude that x is an instance of the class “Travel_History_in_High_risk_area.”

**Fig. 3:**
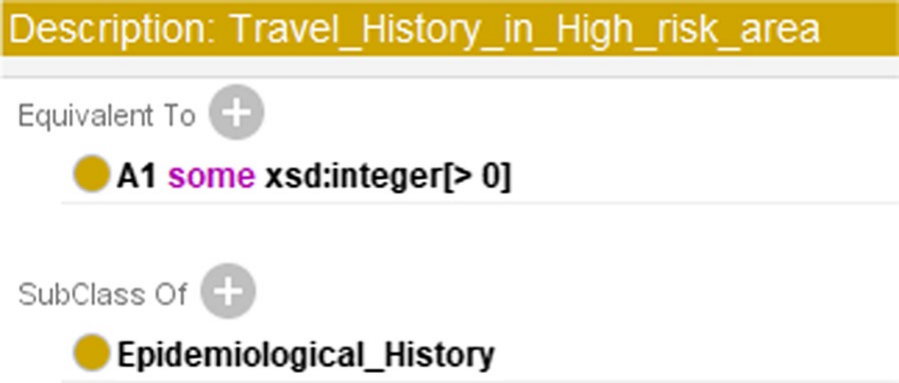
Setting the sufficient and necessary condition for the class “Travel_History_in_High_risk_area.”

Note that for a person, if he has a travel history in a high-risk area, he must either have the data property *A1* as 1, or be an instance of the class “Travel_History_in_High_risk_area.” The two conditions should be set equal. To do this, we set the sufficient and necessary condition for the class “Travel_History_in_High_risk_area” using rule (4), as shown in Fig. 3.

4 $$\text{A1 some xsd: integer} [>0]$$

**Fig. 4:**
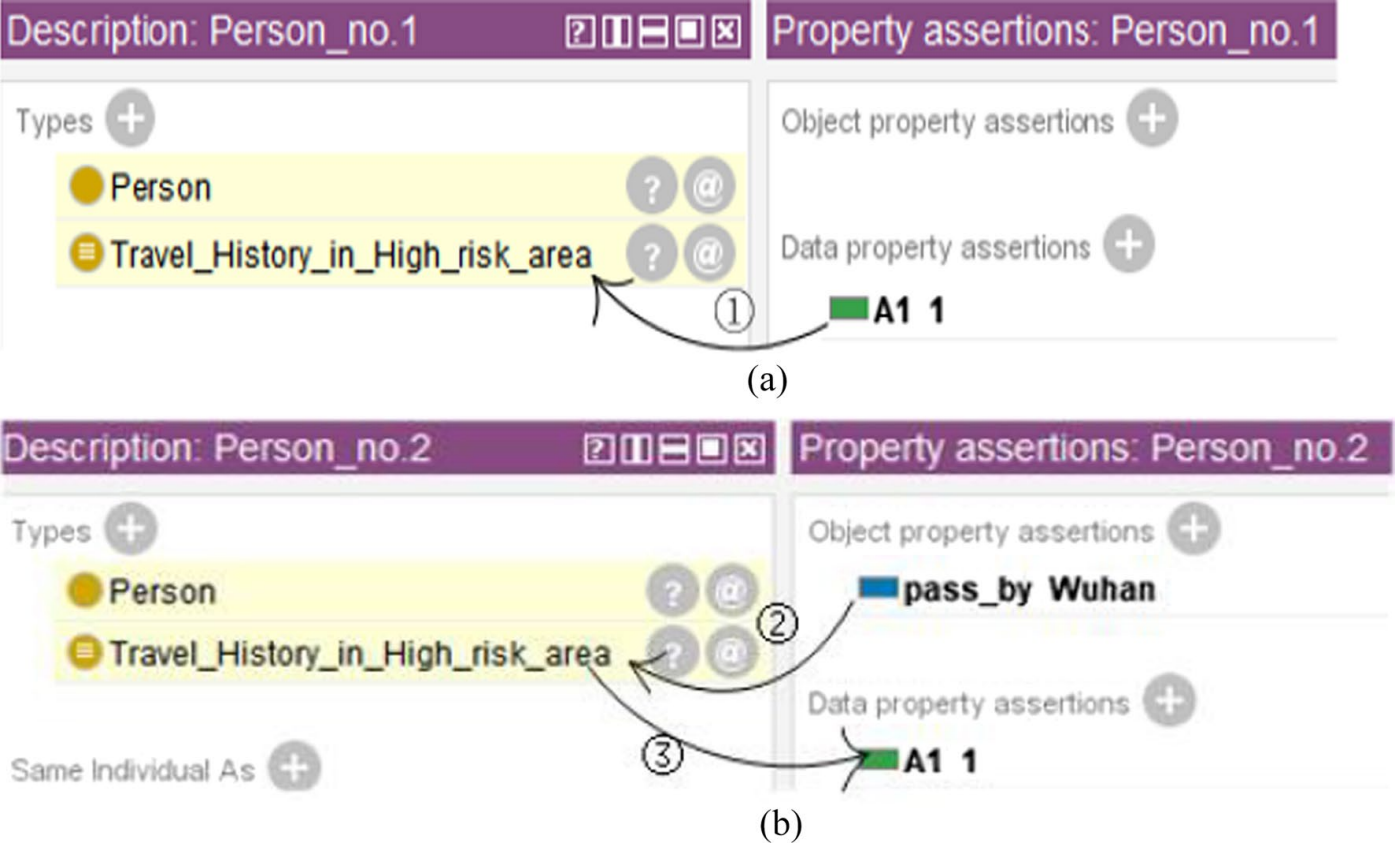
Two ways to indicate that someone has a travel history in a high-risk area: **a** directly assign the data property *A1* to 1. Arrow ① means that Person_no.1 will then automatically be an instance of the class “Travel_History_in_High_risk_area”. **b** Use object property and SWRL rules. The fact that Person_no.2 passed by Wuhan and rule (3) made him an instance of the class “Travel_History_in_High_risk_area.” (Arrow ②). Arrow ③ means that the equivalent condition will then set the data property *A1* to 1

The two ways of indicating that someone has *A1* as an epidemiological history are shown in Fig. 4. One way is by directly assigning the data property *A1* to 1(See Fig. 4a) and the other way is by leveraging the object property *pass_by* and rule (3) (See Fig. 4b).

In the same line of rational, *residence history in a high-risk area(A2)* can also be achieved using data property or object property between class instances.

Next, we take *A4* as an example to demonstrate the methods to model contact histories *A3*, *A4,* and *A5.* As described above, there also exist two scenarios. We can directly assign value 1 to the data property *A4* if we do not know the specific patients our targeted person had contacted. For the other scenario, we use the following SWRL rule (5) to decide if someone contacted a patient who has a fever:

5 $$\text{contact}\_\text{with}(?\text{x}, ?\text{y} )\wedge\text{Fever}\_\text{Symptom}(?\text{y})->\text{Contact}\_\text{with}\_\text{Patients}\_\text{with}\_\text{Fever}\_\text{Symptom}(?\text{x})$$

In the above rule, *contact_with* is an object property between instances of “Person.” We make a hypothesis that every people has contact with his spouse, parents, children, and siblings in this study. As a result, relationships defined in the class “Social_Network” can be used to deduce contacts between people. For instance, rule (6) below indicates that everyone has contact with his spouse.

6 $$\text{hasSpouse}(?\text{x},?\text{y})->\text{contact}\_\text{with}(?\text{x},?\text{y})$$

Also, the same as shown in Fig. 3, the two ways of deciding whether a person has the property *A4* can be set equivalent by giving the equivalent condition for the class “Contact_with_Patients_with_Fever_Symptom.” The modeling of *contact with patients with positive nucleic acid tests(A3)* and *contact with patients with a respiratory symptom(A5)* are similar. Totally, we created 9 SWRL rules for capturing epidemiological histories. For the property *clustering onset(A6),* we only used data property to model it.

#### Modeling of “Clinical Manifestation” and “Etiological or Serological Examination”

As stated in the document [3], clinical manifestations include 3 types:

1. Presenting with fever and/or respiratory symptoms.

2. With chest imaging features of COVID-19 pneumonia.

3. In the early stage of the disease, the total number of leukocytes was normal or decreased, and the lymphocyte count was normal or decreased.

Also, etiological or serological examinations include 3 types:

1. Real-time RT-PCR detection is positive for COVID-19 nucleic acid.

2. The viral gene identified by gene sequencing is highly homologous with known COVID-19.

3. The COVID-19-specific IgM and IgG antibodies are tested positive. The titer of COVID-19-specific IgG antibody is 4 times higher in the recovery period than that in the acute phase.

As the above clinical manifestations and examination results can be achieved directly, we only leveraged data properties to capture them in CDO. The data properties we added for clinical manifestations are: *fever symptom(B1), respiratory symptom(B2), imaging feature(B3), leukocyte count decreased(B4), lymphocyte count decreased(B5)*, and the data properties we added for etiological or serological examinations are: *nucleic acid detection(C1), viral gene sequencing(C2), IgM antibody(C3), IgG antibody(C4).* Each data property takes a value of 0 or 1, where 0 represents a negative result and 1 represents a positive result.

As “Travel_History_in_High_risk_area” in rule (3), for each data property mentioned, we would use it to create a corresponding subclass under the class “Diagnostic_Criteria.” Every person whose data property is positive would be an instance of the corresponding class (see Appendix Table 4).

#### Diagnostic Rules for classes “Suspected_Case” and “Confirmed_Case”

As defined [3], a suspected case of COVID-19 infection is a case that (1) meets any one of the epidemiological history criteria and any two of the clinical manifestations, or (2) demonstrates 3 of the clinical manifestations. A confirmed case of COVID-19 infection is a suspected case with one type of etiological or serological examination.

To determine whether a patient is a suspected case or a confirmed case, it is necessary to check the diagnostic conditions that the patient meets. For instance, to express the condition “meets any one of the epidemiological history criteria,” the restriction rule can be written as follows:
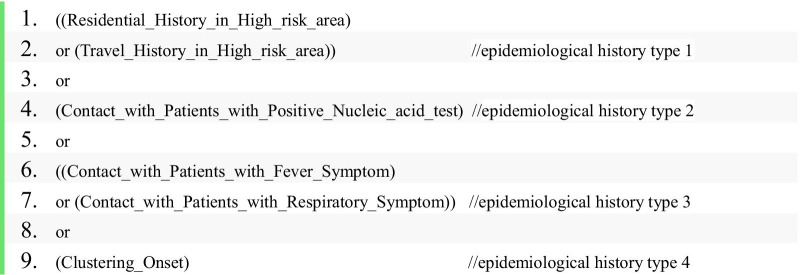


In the above restriction rule, we can also replace the class names with their corresponding data properties because the two ways of obtaining *A1* were already set equivalent (see Fig. 3).

**Fig. 5:**
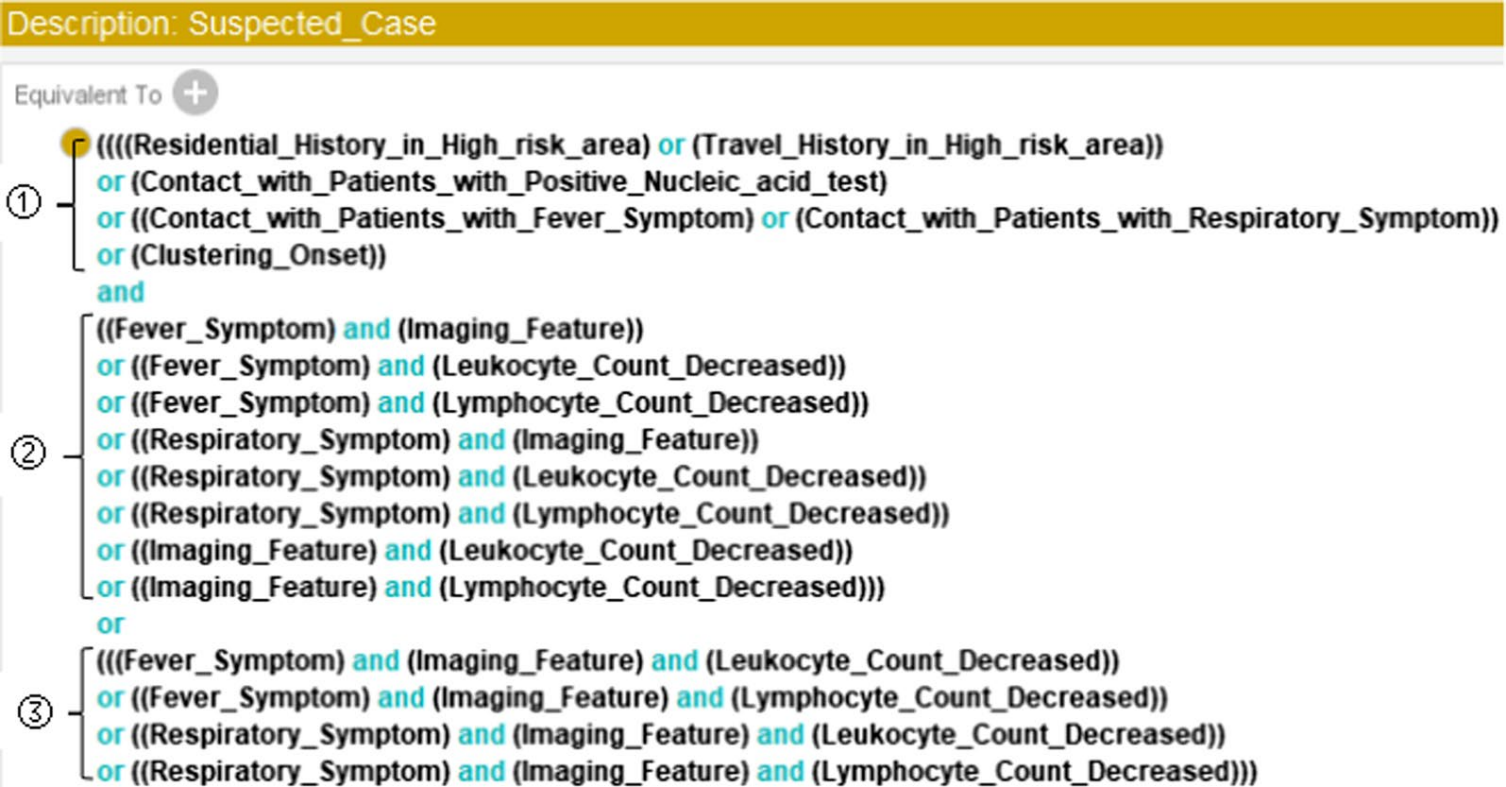
Necessary and sufficient condition for the class “Suspected_Case.” ① represents “meets any one type of epidemiological history,” ② represents “meets any two types of clinical manifestations,” and ③ represents “meets all three types of clinical manifestations.” In short, (① and ②) or ③ is the condition to decide whether the target is a suspected case of COVID-19 infection

According to the diagnosis criteria for suspected cases, the complete sufficient and necessary condition we set for a suspected case in Protégé is shown in Fig. 5.

**Fig. 6:**
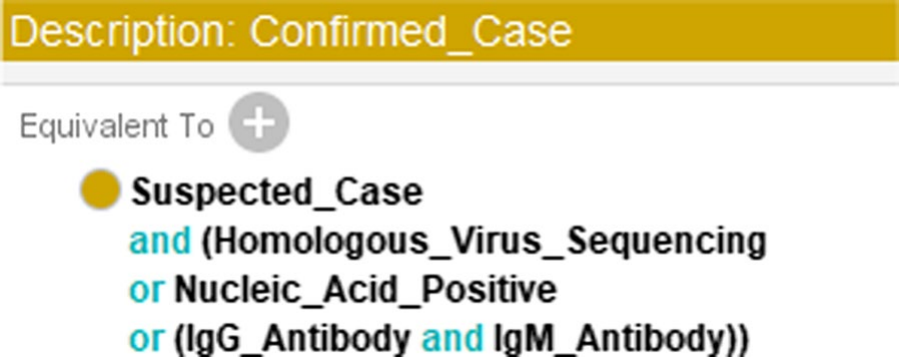
The rule added to “Suspected_Case” to form the sufficient and necessary condition for “Confirmed_Case.”

To define the class “Confirmed_Case,” except for the condition in Fig. 5, we added another condition that represents “meets one type of etiological or serological examination,” as shown in Fig. 6.

### Case data preprocessing

After the classes “Suspected_Case” and “Confirmed_Case” were properly defined, we inputted real patient data into Protégé and verified the automatic diagnosis of the functionality of CDO. We collected the epidemiological survey data of COVID-19 patients in Ningbo City, Zhejiang Province from the Health Commission of Ningbo from 2020.02 to 2020.03 [7] (hereinafter referred to as Case data). Totally there exist 111 pieces of patient data. Without loss of generality, we selected 2 pieces of data for a detailed description. The original data is shown in Table 1. As can be seen from the table, the case data are generally un-structured but formatted data. The main content includes the patient’s basic information, pneumonia symptoms, and imaging features, etc.

**Table 1:** Partial original case data of COVID-19 patients

ID	基本信息	活动事件描述
ID	Basic Information	Event Description
患者1	男, 65岁, 现住海曙区	确诊病例密切接触者。2020 年1月22日发病, 体温38℃, 肺部有影像学改变。现在定点医疗机构隔离治疗。
Patient_no.1	Male 65 years old Live in Haishu District	Close contacts of a confirmed case Onset on January 22, 2020, Body temperature 38 ℃, There are imaging changes in the lungs Now designated medical institutions are isolated for treatment
患者2	男, 56岁, 现住慈溪市	2020年1月14日从武汉回甬, 1月22日发病, 体温38.1℃, 肺部有影像学改变。现在定点医疗机构隔离治疗。
Patient_no.2	Male 56 years old Live in Cixi City	Back to Ningbo from Wuhan on January 14, 2020, Onset on January 22, Body temperature 38.1 ℃, There are imaging changes in the lungs Now designated medical institutions are isolated for treatment

To enable the real cases to be translated into instances recognizable by Protégé, it is necessary to retrieve the properties of each patient. In this study, we used Python language for data preprocessing. Specifically, we leveraged regular expressions and the two functions *compile()* and *findall()* in the *re* module. Table 2 shows the structured case data after preprocessing.

**Table 2:** Structured case data after preprocessing

ID	性别	年龄	住址	症状		
ID	Gender	Age	Address	Symptom		
患者1	男	65	海曙区	确诊病例密切接触者 (核酸检测阳性患者接触)	38 ℃ (发热症状)	肺部有影像学改变 (影像学特征)
Patient_no.1	Male	65	Haishu District	Close contacts with a confirmed case (*A3*)	38 ℃ (*B1*)	There are imaging changes in the lungs(*B3*)
患者2	男	56	慈溪市	武汉回甬 (高风险地区旅行史)	38.1 ℃ (发热症状)	肺部有影像学改变 (影像学特征)
Patient_no.2	Male	56	Cixi City	Back to Ningbo from Wuhan (*A1*)	38.1 ℃ (*B1*)	There are imaging changes in the lungs(*B3*)

The data of Patient_no.1 shows that he meets *A3* as an epidemiological history. Patient_no.2 has *A1* for he returned to Ningbo from Wuhan, and Wuhan belongs to a high-risk area. For both patients, the body temperatures are higher than normal, and there are imaging changes in their lungs, so both have clinical manifestations *B1* and *B3*.

The structured case data can be imported into Protégé in batches through the built-in module Cellfie [23] by the use of transformation rules, which leverage a domain-specific language (DSL) [24] to define the mappings from spreadsheet content to OWL ontology. After data was successfully imported, we could use the defined rules and reasoner in CDO to perform automatic diagnosis.

## Results

The COVID-19 Diagnosis Ontology (CDO) constructed in this study contains 407 concepts, 70 object properties, and 192 data properties. After the construction of CDO, as mentioned earlier, we selected two pieces of representative data from the Ningbo Health Committee [7] for experimental verification.

**Fig. 7:**
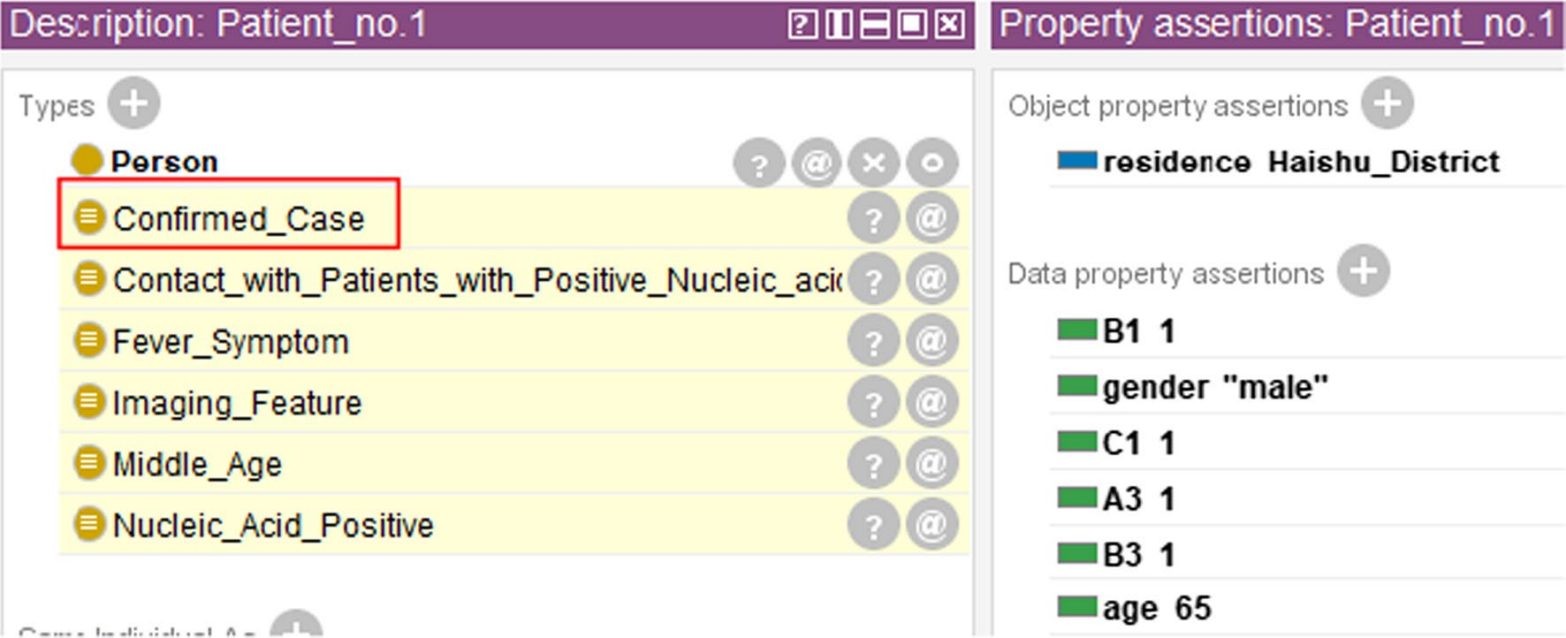
Screenshot of Patient_no.1’s inference results. The manual addition of C1 turned Patient_no.1 into a confirmed case, which was originally a suspected case

**Fig. 8:**
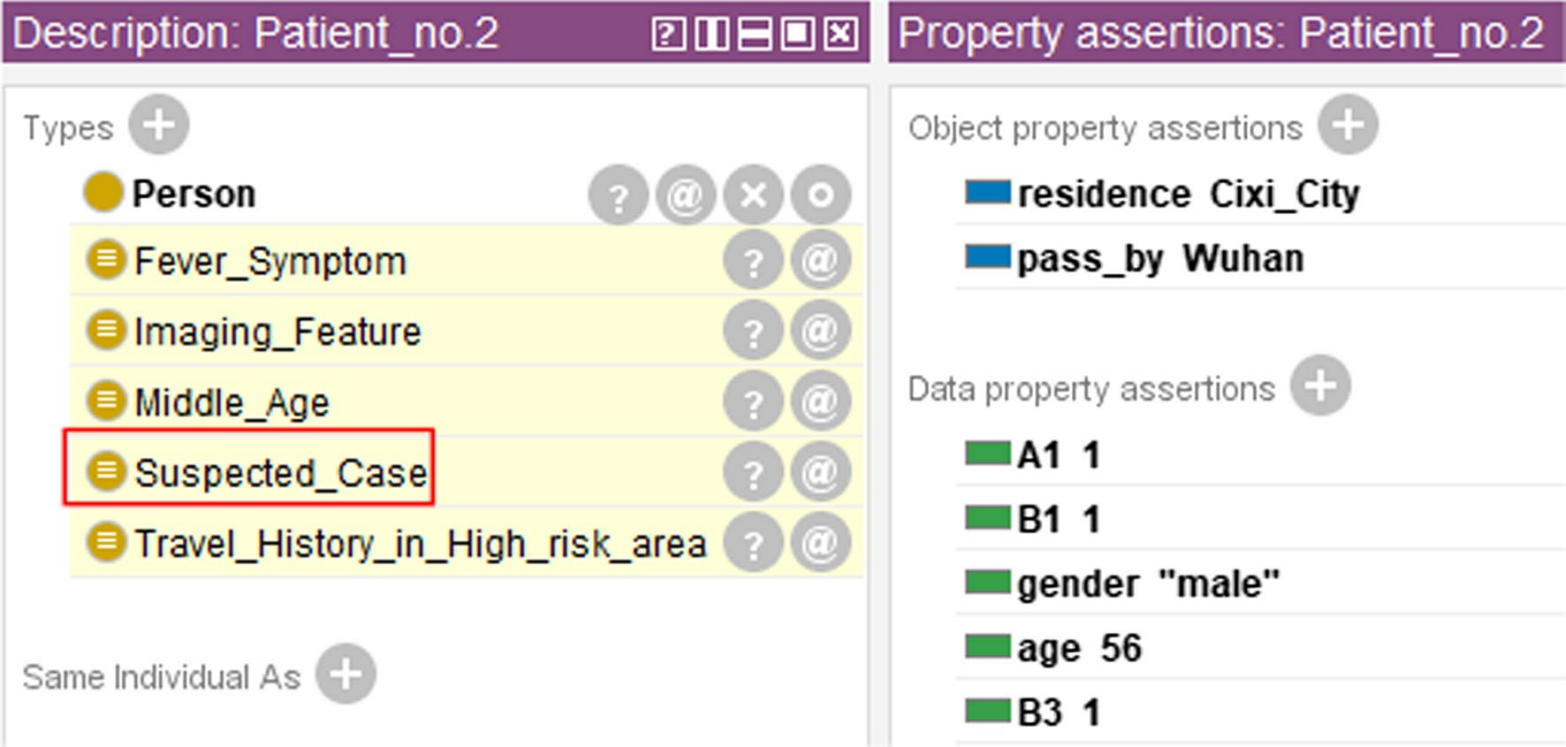
Screenshot of Patient_no.2’s inference results. It can be inferred that Patient_no.2 is a suspected case through his case data

For Patient_no.1, as can be seen from Table 2, he had “close contacts with a confirmed case,” which means that the data property *A3* of him is 1. For Patient_no.2, we have the information that he passed by Wuhan, and Wuhan belongs to high-risk areas, so Patient_no.2 has a positive *A1,* which was obtained by deduction of SWRL rule (3). In addition, both patients have clinical manifestations *B1* and *B3*. After preprocessing and importing the data, the above information was automatically captured as corresponding properties. Then, we started reasoning using Pellet. Figures 7 and 8 show the results after inference.

Note that in reality, both Patient_no.1 and Patient_no.2 are confirmed cases of COVID-19 infection. However, in CDO, we can only deduct that they are suspected cases. The reason is that etiological or serological examination results were missing in the description of the two patients, while this information is required in the diagnostic criteria for “Confirmed_Case.” To test the functionality of the automatic diagnosis module for confirmed cases, we manually set the value of property *C1* as positive for Patient_no.1. The result shows that CDO immediately recognized him as a confirmed case (see Fig. 7). We also tested on the other two types of etiological or serological examinations separately and both results are promising.

## Discussion

In this paper, we proposed a COVID-19 Diagnosis Ontology (CDO) that provides the functionality to automatically decide whether an imported instance is a suspected case or a confirmed case of COVID-19 infection, which is a very important aid for medical workers to quickly detect patients and prevent the spread of the epidemic. Moreover, the knowledge structure we designed for patients can help scholars and researchers in tasks such as data mining and data analysis.

CDO can be used as a tool to filter suspected candidates, which plays an important role when large-scale patient screening is required. For example, a large number of people need to be tested for nucleic acid once a confirmed case occurs, but the results are usually not immediately available. At this time, CDO can screen out suspected cases based on the patient’s epidemiological history, fever, and contact with the confirmed case, etc. For scenarios with massive patient data, CDO can help quickly detect infected patients and greatly reduce labor costs. Besides, the methodology proposed in this paper has a certain reference significance for the unforeseen epidemic in the future.

In addition to the case data in Ningbo, we also collected case data in Zhengzhou from Zhengzhou Municipal Health Commission [25] for experimental verification. In this paper, we show the inference results of two case data, although the cases are not many, experimental results are still sufficient to demonstrate the feasibility of CDO in determining suspected and confirmed cases of COVID-19 infection automatically.

After the outbreak of the 2019-nCoV, the National Health Commission [4] released the “Chinese Clinical Guidance for COVID-19 Pneumonia Diagnosis and Treatment.” By analyzing the epidemic situation, experts have made timely revisions to the diagnosis and treatment plan several times. The CDO created in this study is in accordance with the 7th edition issued in March, and the real patient data we used was also collected in the same time period. To make CDO suitable for later editions, only minor adjustments have to be made as the main framework was already provided. Future research may focus on the following aspects:

The COVID-19 Diagnosis Ontology (CDO) can successfully diagnose suspected and confirmed cases of infection based on the properties imported into Protégé. Nevertheless, it is not convenient for medical staff and researchers to use, so a visual query interface will be built subsequently to facilitate data management and inquire. More specifically, by inputting unstructured case data, the query system can automatically diagnose whether the patient is a suspected or a confirmed case after analysis, rather than the current structured case data.

The reasoning rules can be more detailed and cover the temporal and spatial activities of the population. For one instance, contacts between people are limited to the contacts between relatives in this study. In the future, we will include contacts that happened by taking the same means of transportation, or having been to the same place at the same time, etc. For another instance, the risk levels of different areas are changing dynamically. Later, it is feasible to use the current number of infected patients to judge if a place is a high-risk area. By capturing and analyzing much detailed information, we can better prevent the spread of COVID-19.

Although CDO can calculate the number of COVID-19 infections in different age groups, due to data integrity limitations, it is a great challenge to make use of it in this study. While the enormous number of case data is collected in the follow-up study, this functionality can be leveraged to analyze the susceptibility of different age groups to COVID-19, and to take different prevention and control measures for different age groups, so as to control COVID-19 more efficiently.

## Conclusions

The outbreak of the 2019-nCoV has severely affected human health and social stability, and research on COVID-19 has become a hot spot today. In this paper, we built the COVID-19 Diagnosis Ontology (CDO) based on the conceptual support of COKG-19 and FOAF. More importantly, we modeled the diagnosis criteria of COVID-19 in CDO by the creations of classes, data properties, object properties, and SWRL rules, so that the automatic diagnosis of confirmed and suspected cases of COVID-19 infection from real-case data is made possible. Summarizing this paper, CDO will not only significantly reduce the manual input in the diagnosis process of COVID-19, but also uncover hidden cases and help prevent the widespread of this epidemic.

## Data Availability

The dataset supporting the conclusions of this article is available from the corresponding author upon reasonable request.

## Appendix

See Tables 3 and 4.

**Table 3:** Some SWRL rules in CDO

Number	Category	Name	SWRL Rules
1	Social_Network	Parents_Relationship1	Person(?y)^hasParent(?x,?y)^hasSpouse(?y,?z)- > hasParent(?x,?z)
2		Parents_Relationship2	hasParent(?x,?y)^hasSibling(?x,?z)- > hasParent(?z,?y)
3		Father_Relationship1	hasParents(?x, ?y) ^ Male(?y)—> hasFather(?x, ?y)
4		Father_Relationship2	hasFather(?x,?y)^hasSpouse(?x,?z)—> hasFather(?z, ?y)
5		Father_Relationship3	hasSibling(?x,?y)^hasFather(?x,?z)- > hasFather(?y,?z)
6		Mother_Relationship1	hasParents(?x, ?y) ^ Female(?y)—> hasMother(?x, ?y)
7		Mother_Relationship2	hasMother(?x,?y)^hasSpouse(?x,?z)—> hasMother(?z, ?y)
8		Mother_Relationship3	hasSibling(?x,?y)^ hasMother (?x,?z)- > hasMother (?y,?z)
9		Children_Relationship1	hasChildren(?x,?y)^hasSpouse(?y,?z)- > hasChildren(?x,?z)
10		Children_Relationship2	hasChildren(?x,?y)^hasSpouse(?x,?z)- > hasChildren(?z,?y)
11		Son_Relationship1	Person(?x) ^ hasChildren(?x, ?y) ^ Male(?y)—> hasSon(?x, ?y)
12		Son_Relationship2	hasChildren(?x,?y)^Male(?y)^hasSpouse(?x,?z)- > hasSon(?z,?y)
13		Daughter_Relationship1	Person(?x)^hasChildren(?x,?y)^Female(?y)- > hasDaughter(?x, ?y)
14		Daughter_Relationship2	hasChildren(?x,?y)^Female(?y)^hasSpouse(?x,?z)- > hasDaughter (?z,?y)
15		Brother_Relationship	Person(?x)^hasSibling(?x,?y)^Male(?y)- > hasBrother(?x, ?y)
16		Sisters_Relationship	Person(?x)^hasSibling(?x,?y)^Female(?y)- > hasSister(?x, ?y)
17		Aunt_Relationship	Person(?x)^hasParents(?x,?y)^hasSister(?y,?z)- > hasAunt(?x, ?z)
18		Uncle_Relationship	Person(?x)^hasParents(?x,?y)^hasBrother(?y,?z)- > hasUncle(?x, ?z)
19		Nephew_Relationship1	hasAunt(?x, ?y) ^ Male(?x)—> hasNephew(?y, ?x)
20		Nephew_Relationship2	hasUncle(?x, ?y) ^ Male(?x)—> hasNephew(?y, ?x)
21		Nephew_Relationship3	hasSibling(?x,?y)^hasSon(?y,?z)- > hasNephew(?x, ?z)
22		Niece_Relationship1	hasUncle(?x, ?y) ^ Female(?x)—> hasNiece(?y, ?x)
23		Niece_Relationship2	hasAunt(?x, ?y) ^ Female(?x)—> hasNiece(?y, ?x)
24		Niece_Relationship3	hasSibling(?x,?y)^hasDaughter(?y,?z)- > hasNiece(?x, ?z)
26	Epidemiological_History	Contact_History1	contact_with(?x,?y)^Fever_Symptom(?y)- > Contact_with_Patients_with_Fever_Symptom(?x)
27		Contact_History2	contact_with(?x,?y)^Respiratory_Symptom(?y)- > Contact_with_Patients_with_Respiratory_Symptom(?x)
28		Contact_History3	contact_with(?x,?y)^Nucleic_Acid_Positive(?y)- > Contact_with_Patients_with_Positive_Nucleic_acid_test(?x)
29		Contact_History4	hasSpouse(?x,?y)- > contact_with(?x,?y)
30		Contact_History5	hasChild(?x,?y)- > contact_with(?x,?y)
31		Contact_History6	hasSibling(?x,?y)- > contact_with(?x,?y)
32		Contact_History7	hasParent(?x,?y)- > contact_with(?x,?y)
33		Residential_History_in_High_risk_areas	Person(?x)^residence(?x,?y)^high_risk_area(?y)- > Residential_History_in_High_risk_area(?x)
34		Travel_History_in_High_risk_areas	Person(?x)^pass_by(?x,?y)^high_risk_area(?y)- > Travel_History_in_High_risk_area(?x)

**Table 4:** Labels of data properties

Class	Subclass	Data Property	Label
Epidemiological History	Travel_History_in_High_risk_area	Travel history in a high-risk area	A1
	Residential_History_in_High_risk_area	Residence history in a high-risk area	A2
	Contact_with_Patients_with_Positive_Nucleic_acid_test	Contact with patients with positive nucleic acid tests	A3
	Contact_with_Patients_with_Fever_Symptom	Contact with patients with a fever symptom	A4
	Contact_with_Patients_with_Respiratory_Symptom	Contact with patients with a respiratory symptom	A5
	Clustering_Onset	Clustering onset	A6
Clinical Manifestation	Fever_Symptom	Fever symptom	B1
	Respiratory_Symptom	Respiratory symptom	B2
	Imaging_Feature	Imaging feature	B3
	Leukocyte_Count_Decreased	Leukocyte count decreased	B4
	Lymphocyte_Count_Decreased	Lymphocyte count decreased	B5
Etiological or Serological Examination	Nucleic_Acid_Positive	Nucleic acid detection	C1
	Homologous_Virus_Sequencing	Viral gene sequencing	C2
	IgM_Antibody	IgM antibody	C3
	IgG_Antibody	IgG antibody	C4

## Abbreviations

COVID-19: Corona Virus Disease 2019
CDO: COVID-19 Diagnosis Ontology
FOAF: Friend of a Friend
SWRL: Semantic Web Rule Language
